# Clinical application of the anterior pelvic wall locking plate (APWLP) in acetabular fractures involving the quadrilateral surface

**DOI:** 10.1186/s13018-022-03392-y

**Published:** 2023-01-31

**Authors:** ZhiDong Wang, ZhenHeng Wang, GuangDong Chen, MaoFeng Gao, RuoFu Zhu, HuiLin Yang

**Affiliations:** Department of Orthopaedics, The First Affiliated Hospital of Soochow University, 899 Pinghai Road, Suzhou, 215006 China

**Keywords:** Acetabular fracture, Lateral rectus, Quadrilateral surface

## Abstract

**Background:**

The management of acetabular quadrilateral surface fractures remains challenging for surgeons, and the treatment options for such fractures remain controversial. Quadrilateral surface surgery is a complex procedure involving combined approaches, and the quality of fracture reduction closely depends upon the surgical procedure, as well as the skill and experience of the surgeon. This study aimed to explore the clinical effects of applying an anterior pelvic wall locking plate (APWLP) through the lateral-rectus approach for treating acetabular fractures involving the quadrilateral surface.

**Methods:**

This retrospective analysis was comprised of 35 patients with acetabular fractures involving the quadrilateral surface who were treated with an APWLP in our hospital between June 2016 and December 2020. The patients included 25 males and ten females, with an average age of 52.45 years. All the patients were exposed through the lateral-rectus approach, six patients were exposed with an additional iliac fossa approach, and the fractures were fixed by combining an APWLP with a reconstruction plate. The Matta imaging standard was used to assess the quality of the fracture reduction, and the final follow-up clinical outcome was classified as excellent (18 points), good (15–17), fair (13–14), or poor (< 13) according to the modified Merle d’Aubigné-Postel scoring standard.

**Results:**

All patients successfully completed the operation, and there was no blood vessel or nerve injury during any of the operations. The average follow-up period was 26.11 months. The mean time of resuming full-weight-bearing activities was 12.88 weeks. Hip flexion and extension and internal and external rotation ranges of motion significantly increased over time. At the last follow-up, Matta’s imaging evaluation showed that 24 cases were anatomically reduced, seven cases were satisfactory, and four cases were unsatisfactory. The satisfaction rate was 88.6% (31/35). According to the modified Merle d’Aubigné-Postel scoring standard, the hip function was excellent, good, fair, and poor in 23, 6, 4, and 2 cases, respectively. The excellent and good rates represented 82.9% of the total cases (29/35).

**Conclusion:**

The findings suggest that the APWLP for acetabulum quadrilateral surface fracture achieve good to excellent clinical and radiological outcomes, and an APWLP may be a new treatment option for these fractures involving the quadrilateral surface.

## Background

In 1964, Judet and Letournel characterized the inner wall of the acetabulum as a quadrilateral surface [1]. Except for simple anterior or posterior wall fractures, acetabular fractures involve the quadrilateral surface [2]. The quadrilateral surface is surrounded by a thin part of the hip bone and has an irregular shape and a deep anatomical location adjacent to surrounding essential blood vessels and nerves [3, 4]. Therefore, the surgical management of acetabular fractures in the square zone is highly challenging.

Quadrilateral surface fractures are intra-articular fractures whose management requires an anatomical reduction to stabilize the fracture and restore the correct anatomy of the acetabulum. Otherwise, they can easily cause central dislocation of the hip joint and traumatic arthritis, eventually impairing treatment efficacy [2, 5]. Presently, in the clinical treatment of quadrilateral surface fractures, the anterior approach is often used to expose the fracture, the anterior column is fixed using a reconstruction plate, and the posterior column is fixed using a lag screw or plate through the posterior Kocher-Langenbeck (K-L) approach [6]. Ciolli et al. [7] reported that quadrilateral surface (QLS) plates in association with the anterior intrapelvix (AIP) approach represented an effective treatment strategy for acetabular fractures with anterior involvement. However, it is challenging to fix and block such fractures well via this fixation method [8].

To overcome these problems, our hospital has combined an APWLP with a reconstruction plate in the treatment of quadrilateral surface acetabular fractures since June 2016. Clinical data on the involved patients were retrospectively analyzed, and the clinical results at the early follow-up were satisfactory.

## Equipment introduction

The APWLP (Shandong Weigao Orthopedic Materials Co. Ltd., Shandong, China) is a titanium alloy with an arcuate edge plate and two locking plates. It has a J-shaped integrated structure. The arcuate rim plate includes iliac and pubic holes. The two J-shaped structures are in a cross-structure with a gap between them to observe the reduction.

**Fig. 1:**
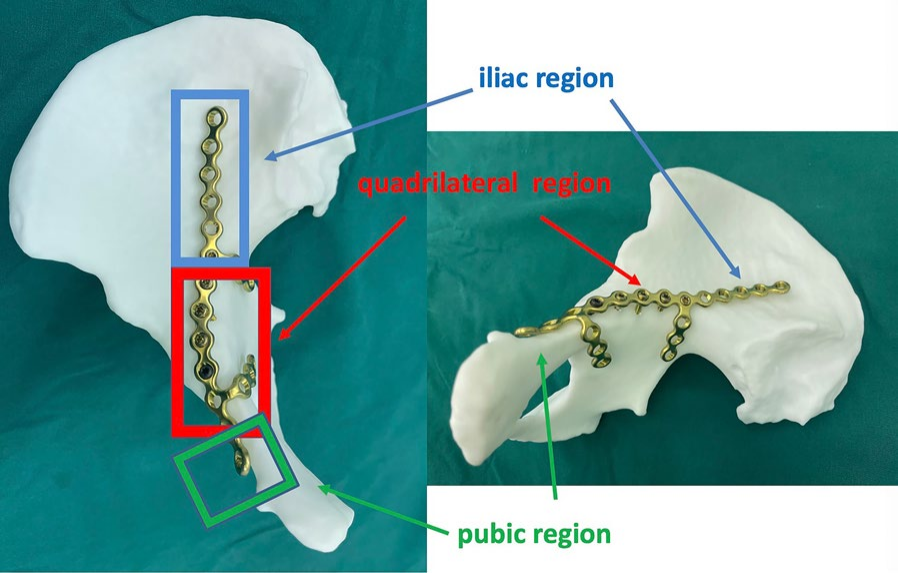
Structure and components of the **APWLP**. It is a titanium alloy with a J-shaped integrated structure, an arcuate edge plate, and two locking plates. The arcuate edge plate is placed on the anterior edge of the acetabulum. The two locking plates are distributed in a J-shape to block or fix the quadrilateral surface. According to the placement trajectory on the pelvis, an **APWLP** was divided into three parts: the iliac region, the quadrilateral region, and the pubic region

**Fig. 2:**
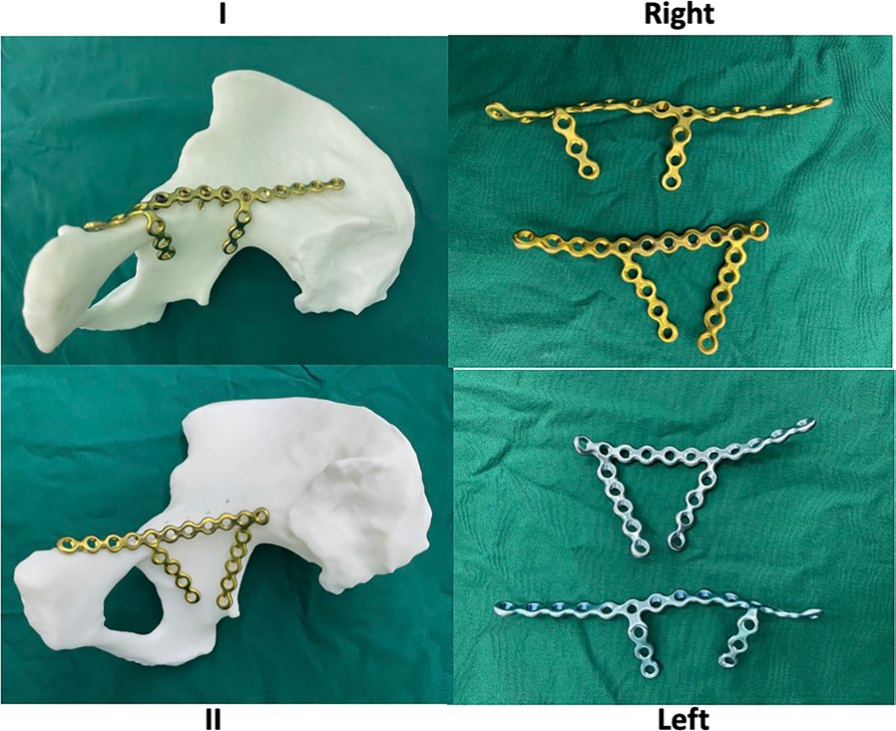
Two different **APWLP** models. Yellow represents the right side, and blue represents the left side. The specific type of steel plate is selected according to the shape of the quadrilateral surface fracture. There are two **APWLP** models: I and II

The J-shaped structure blocks the quadrilateral surface bone from the medial to the outer side. Locking screws are inserted into the nail holes between the J-shaped structures, distributed like a fence to effectively prevent the quadrilateral surface bone from shifting to the medial side, and the J-shaped structure properly blocks the bones at the quadrilateral surface. The locking plate placed in the anterior pelvic wall blocks the bones at the quadrilateral surface from the inside out during surgery, properly stabilizing the acetabular fracture (Figs. 1 and 2).

## Materials and methods

The Ethics Committee of the First Affiliated Hospital of Soochow University approved this study. All the selected patients proved signed informed consent.

### Patient selection criteria

#### Inclusion criteria

1. Patients who underwent surgical treatment for acetabular fractures at the First Affiliated Hospital of Soochow University between June 2016 and December 2020.
2. Patients aged 18–82 years (regardless of gender), with acetabular fractures involving the quadrilateral surface according to the Letournel–Judet classification.
3. Acetabular fractures involving the quadrilateral surface, such as an anterior wall or anterior column with a posterior semi-transverse, T-shaped, double-column, or transverse fracture.
4. Patients with a previously exposed quadrilateral surface who had been treated via a lateral-rectus approach combined with an APWLP approach.
5. Patients with complete data who had been followed up for > 1 year.

#### Exclusion criteria

1. Open fractures of the pelvis or acetabulum.
2. Fractures not treated with an APWLP.
3. Patients with severe medical diseases, such as hepatic or renal insufficiency, hematopoietic diseases, and diseases of the circulatory and respiratory systems, and those who could not undergo surgery.
4. Patients with incomplete medical records or imaging data.

### General information

**Fig. 3:**
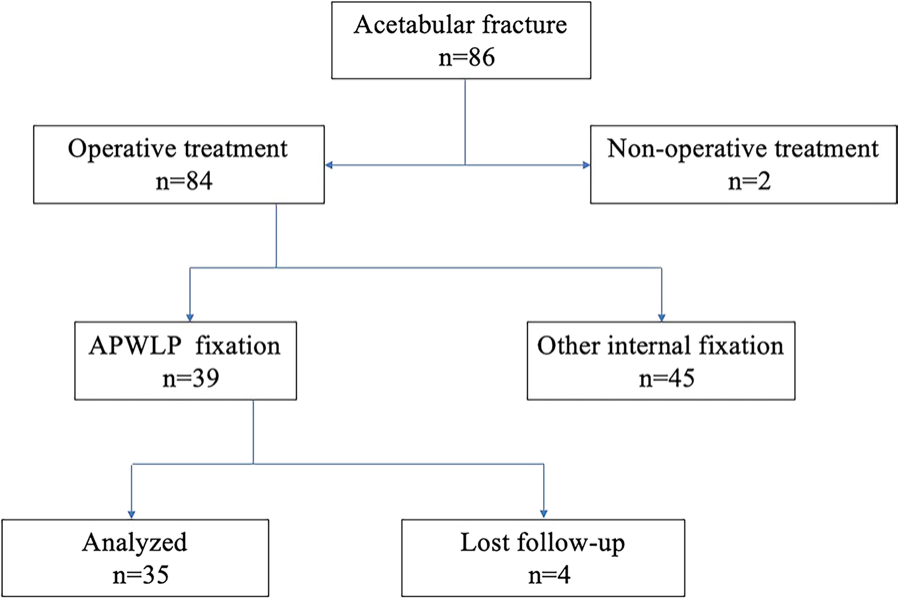
A flowchart of inclusions

From June 2016 to December 2020, 86 patients with acetabular fractures were treated in our hospital. Of the 86 patients, 39 were treated using an APWLP. Out of the 39 patients, one patient lacked complete medical records, and three patients who lacked imaging data were not included in the follow-up. The 35 patients including 25 males and 10 females (23–82 years old; average, 52.45 ± 15.87 years) who met the inclusion and exclusion criteria were available for evaluation (Fig. 3). Fracture classification was based on the Letournel–Judet classification. There were 13 cases of double-column fractures, seven cases of T-shaped fractures, seven cases of anterior wall with posterior semi-transverse fractures, and eight cases of transverse fractures. Among them, 29 cases involved extra-articular pelvic rim fractures. Thirteen cases were injuries from falls (10 from heights of > 2 m and 3 from trips or slip falls), and 22 cases were due to car accidents. The timing of surgery was 2–15 d (average, 5.22 ± 2.70 d).

### Surgical technique

All operations were performed by a chief physician who had completed acetabular fracture surgery through the lateral-rectus approach for more than 200 patients at our hospital.

**Fig. 4:**
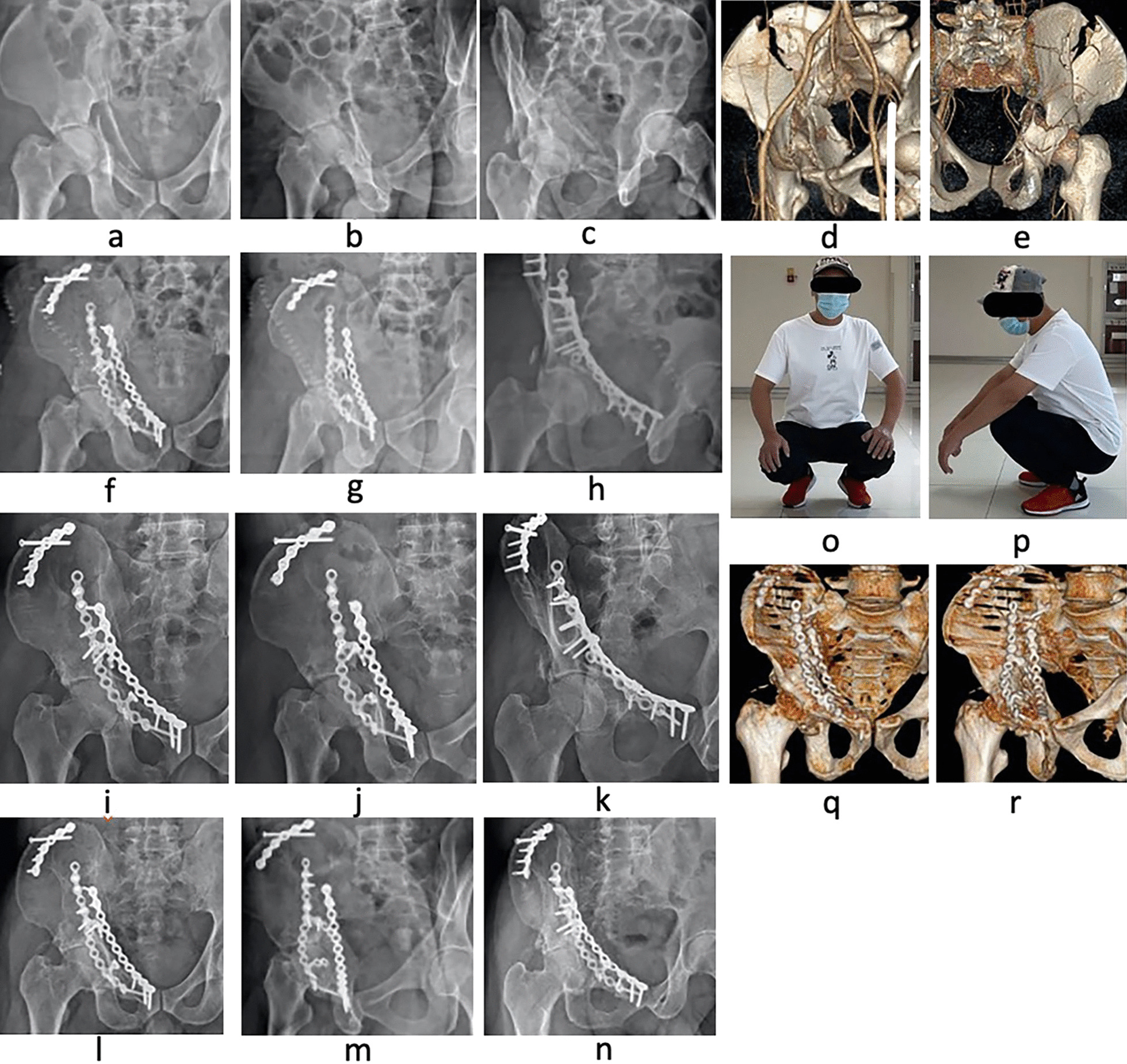
A 44-year-old man presented with an anterior column and posterior hemitransverse fracture of the right acetabulum following a traffic accident. The case involved pelvic fractures Preoperative AP (**a**), iliac oblique position (**b**), obturator oblique position (**c**), and 3D CT reconstruction **d**, **e** of the pelvis confirmed the fracture pattern. On the 7th day after the injury, fixation was performed through the lateral-rectus approach using an **APWLP** combined with a reconstruction plate. Postoperative AP (**f**, **i**, **l**), iliac oblique position (**g**, **j**, **m**), obturator oblique position (**h**, **k**, **n**), and 3D CT reconstruction (**q**, **r**), showing excellent Matta’s X-ray evaluation scores. Modified Merle d’Aubigné evaluation scores were excellent and hip flexion function **o**, **p** was satisfied (**f**, **g**, and **h** are X-rays taken 3 days postoperatively; **i**, **j**, and **k** are X-rays taken 3 months postoperatively; **l**, **m**, **n** are X-rays taken 1 year postoperatively; and **q** and **r** are 3D CT reconstructions done 1 year postoperatively)

First, the patient was placed in the supine position on the operating table to ensure that the C-arm machine could see through the surgical site during the operation. The affected lower extremity was routinely disinfected to facilitate hip flexion surgery and traction to reduce acetabular fractures. Exposure of the belly button and pubic symphysis facilitated the determination of anatomical landmarks during surgery. The lateral-rectus approach was used to expose and fix the fractures. The details of the surgical procedure and operative techniques were previously described by Chen et al. [9]. All of the quadrilateral surface fractures were fixed with an APWLP, and the other fractures were fixed with AO reconstruction plates. The C-arm machine was used once again to assess for fracture reduction and check the position of the plate screw to ensure that the screw did not enter the joint cavity (Fig. 4). The operation time and amount of intraoperative blood loss were routinely recorded.

First-generation cephalosporins were routinely used to prevent postoperative infections within 48 h. Anti-thrombotic pressure bands were applied after surgery to prevent venous thrombosis of the lower extremities. Low-molecular-weight heparin was used for anticoagulation on the first day after surgery. Patients with drainage tubes had the tubes removed when the drainage volume was < 50 mL/d after surgery. The hip joint was actively and passively moved on the second day after surgery, and a walker was used to perform functional exercises within 6 weeks after surgery.

### Radiographic and clinical evaluation

All patients underwent three standard plain radiographic examinations, namely for the anteroposterior pelvis and obturator and iliac oblique views, before surgery; 1, 3, 6, and 12 months after surgery; and at the last follow-up. All patients underwent three-dimensional (3D) pelvic computed tomography before surgery. Fractures were classified according to the Letournel–Judet classification.

The quality of fracture reduction was evaluated according to the Matta imaging standard. A fracture displacement of < 1 mm implied anatomical reduction, 1–3 mm implied satisfactory reduction, and > 3 mm implied unsatisfactory reduction.

Clinical outcomes were evaluated according to the modified Merle d’Aubigné-Postel scoring standard. The score evaluation involves three aspects: pain, walking, and range of joint motion. Scores of 18, 15–17, 12–14, and 3–11 points are considered excellent, good, fair, and poor, respectively.

### Statistical methods

Statistical analyses were performed using the SPSS 23.0 (SPSS, USA) statistical software, and the data obtained are presented as $\overline{x} \pm s$. For normally distributed data, a paired sample *t* test was performed. The enumeration data were tested using Chi-squared or Fisher's exact test. *P* < 0.05 was considered to indicate statistical significance.

## Results

All 35 patients successfully completed the operation. The peritoneum of three patients was ruptured when the tissue was separated due to severe adhesions between the peritoneum and the surrounding soft tissue, and the rupture was immediately sutured. The timing of surgery was 2–15 d (average, 5.22 ± 2.70 d). Femoral blood vessels, femoral nerve, spermatic cord, uterine round ligament, and obturator nerve were not injured during surgery. The operation time was 120–450 min (average, 245.14 ± 65.32 min), and the amount of intraoperative blood loss was 300–2100 mL (average, 825.71 ± 429.32 mL). The postoperative incision drainage volume was 50–180 mL (average, 105.85 ± 33.57 mL). The incisions of all patients healed well without complications, such as hematomas, fat liquefaction, exudation, and swelling. Deep vein thrombosis occurred in two patients after surgery. There were no complications, such as femoral head necrosis or early traumatic arthritis, at the last follow-up.

The follow-up time of the 35 patient who matched the inclusion criteria was 12–42 months (average, 26.11 ± 10.53 months). None of the patients presented signs of non-union of bone fragments 8–22 weeks (average, 13.02 ± 3.33 weeks) after surgery. The full-weight-bearing activity time of the patients was 8–24 weeks (average, 12.88 ± 4.31 weeks). The postoperative hip flexion and extension and internal and external rotation range of motion were significantly higher at the last follow-up than at 3 months after surgery (127.85 ± 12.08 and 52.14 ± 7.20 vs. 106.42 ± 12.03 and 38.00 ± 8.15, respectively; *P* < 0.001). According to the modified Merle d’Aubigné-Postel scoring standard, the hip function was excellent in 23 cases, good in six cases, fair in four cases, and poor in two cases. Excellent and good rates were achieved in 82.9% of the total cases (29/35).

At the last follow-up, none of the patients had any complications, such as screw loosening, prolapse, femoral head necrosis, and heterotopic ossification. Matta’s imaging evaluation showed that 24 cases were anatomically reduced (68.6%, 24/35), seven cases were satisfactory (20%, 7/35), and four cases were unsatisfactory (11.4%, 4/35). The satisfaction rate was 88.6% (31/35). There were four and six cases of hip space stenosis 3 months after surgery and at the last follow-up, respectively. However, the difference was not statistically significant (*P* = 0.734).

## Discussion

Quadrilateral surface fractures often involve the anterior and posterior acetabulum columns, and the traditional anterior and posterior approaches using reconstruction plate fixation are unsatisfactory [6, 8]. Several studies have reported that it was difficult to obtain high stability in such fractures using a traditional plate or screw fixation, generally resulting in fixation failure and poor clinical outcomes [10–12]. Therefore, we tested the efficacy of an APWLP in treating these fractures. We obtained satisfactory clinical and imaging results, providing a new internal fixation option for treating acetabular quadrilateral surface fractures.

The choice of internal fixation is critical for maintaining the quality of acetabular fracture reduction. The key to surgical treatment is to prevent inward or downward displacement of the quadrilateral surface fracture. Robin [13] recommended the use of a buttress plate to control the medial displacement of the quadrilateral surface through the ilioinguinal approach and reported good results in 85% of the patients. Sen et al. [14] reviewed the application of an anatomical quadrilateral plate for acetabular quadrilateral surface fractures. Anatomical reductions were obtained in 28 of 33 patients. This approach provides a good outcome for elderly patients. Farid et al. [2] used a cerclage wire plate composite for fixing acetabular quadrilateral plate fractures and reported satisfactory clinical outcomes. However, for comminuted fractures of the square area of the acetabulum, this fixation method is not reliable and is prone to the loss of reduction and failure. Additionally, the usage of a cerclage wire or cable, which needs to pass through the greater sciatic notch, to fix the fracture poses a high risk of injury to the sciatic nerve or superior gluteal vessel. Furthermore, these fixation techniques require a surgeon with a wealth of skills and experience.

The reduction quality in acetabular fractures is closely related to surgical exposure [15]. The traditional ilioinguinal approach for treating acetabular, anterior column fractures was first reported by Letournel in 1993 [14]. The modified Stoppa approach [16, 17] is suitable for exposing and fixing quadrilateral surface fractures, but not in severely obese patients or those with well-developed abdominal muscles. Both surgical approaches can damage blood vessels and nerves, and the learning curve is long due to the presence of important anatomical structures in the surgical field. The use of the pararectus approach to treat acetabular fractures was first reported by Keel et al. in 2012 [18]. According to the Letournel–Judet classification, this surgical approach is suitable for treating patients with acetabular fractures that do not involve the posterior wall of the acetabulum. In our study, we exposed the fracture using the lateral-rectus approach, which could directly reveal the quadrilateral surface, arcuate edge, and sacroiliac joint. Then, we used an APWLP to fix the quadrilateral surface fracture. In comminuted fractures, a J-shaped APWLP and fence screw were used for blocking.

In our study, none of the patients had screw loosening, prolapse, femoral head necrosis, or heterotopic ossification. Excellent and good success rates were achieved in 82.9% of the cases based on the modified Merle d’Aubigné-Postel scoring system. Matta’s imaging evaluation showed a 68.6% anatomical reduction. The clinical outcomes and rates of the anatomical reduction in the quadrilateral surface fracture are similar between this study and previous studies [5, 19–21], but the patients in our study had a smaller surgical incision, less blood loss, lower rates of loss reduction, and fewer complications than the patients in the previous studies. Therefore, we believe that an APWLP has the advantage of being a simple and highly safe option.

We treated acetabular quadrilateral surface fractures using an APWLP through the lateral-rectus approach. The follow-up results were satisfactory, with good hip joint function overall. However, there were some limitations to this study. Firstly, it was based on retrospective data analysis and lacked a control group. There were some potential biases such as surgeon experience and patient selection. However, we consider the results valuable because they provide a new technique for acetabular fractures involving the quadrilateral surface. In addition, further and larger-sized studies should be conducted to determine whether the results are comparable to or better than the results after the application of standard implants. Secondly, the sample size was small due to the low incidence of acetabular fractures involving the quadrilateral surface. Studies with larger sample sizes and randomized controlled trials should be performed in the future to confirm our conclusions. Thirdly, although computed tomography (CT) scans can better assess the quality of reduction, we used X-rays to determine reduction quality postoperatively. Because CT scans are too expensive for some poor patients, some teams suggest that CT evaluations are only beneficial for some fractures with inconclusive or definite malpositioning of the implants on perioperative or postoperative radiographs [22].

## Conclusion

In conclusion, APWLP is a simple technique for acetabular fractures involving the quadrilateral surface. In terms of clinical and radiological outcomes, APWLP and other plate have similar results. However, APWLP has the advantage of smaller surgical incision, less blood loss, lower rates of loss reduction, and an APWLP may be a new treatment option for these fractures involving the square area of the acetabulum.

## Data Availability

The datasets generated and/or analyzed during the current study are not publicly available due to continuing research using the data. However, they are available from the corresponding author upon reasonable request.

## Abbreviations

APWLP: Anterior pelvic wall locking plate
CT: Computed tomography
CTA: Computed tomography artery

## References

[1] Judet R, Judet J, Letournl E (1964). Fracture of the acetabulum: classification and surgical approaches for open reduction. Preliminary report. J Bone Jt Surg Am.

[2] Farid YR (2010). Cerclage wire-plate composite for fixation of quadrilateral plate fractures of the acetabulum: a checkrein and pulley technique. J Orthop Trauma.

[3] Henry PDG, Kreder HJ, Jenkinson RJ (2013). The osteoporotic acetabular fracture. Orthop Clin N Am.

[4] Ferguson TA, Patel R, Bhandari M (2010). Fractures of the acetabulum in patients aged 60 years and older: an epidemiological and radiological study. J Bone Jt Surg Br.

[5] Laflamme GY, Hebert-Davies J, Rouleau D (2011). Internal fixation of osteopenic acetabular fractures involving the quadrilateral plate. Injury.

[6] Tosounidis TH, Gudipati S, Panteli M (2015). The use of buttress plates in the management of acetabular fractures with quadrilateral plate involvement: is it still a valid option?. Int Orthop.

[7] Ciolli G, Mauro D, Rovere G (2021). Anterior intrapelvic approach and suprapectineal quadrilateral surface plate for acetabular fractures with anterior involvement: a retrospective study of 34 patients. BMC Musculoskelet Disord.

[8] Bastia JD, Savic M, Cullmann JL (2016). Surgical exposures and options for instrumentation in acetabular fracture fixation: pararectus approach versus the modified Stoppa. Injury.

[9] Chen JH, Liu H, Wang CB (2019). Internal fixation of acetabular fractures in an older population using the lateral-rectus approach: short-term outcomes of a retrospective study. J Orthop Surg Res.

[10] White G, Kanakaris NK, Faour O (2013). Quadrilateral plate fractures of the acetabulum: an update. Injury.

[11] Jang CY, Kwak DK, Lee HM (2019). Management of anteromedially displaced acetabular fractures using a collinear reduction clamp through modified ilioinguinal approach. Orthop Traumatol Surg Res.

[12] Boudissa M, Francony F, Kerschbaumer G (2017). Epidemiology and treatment of acetabular fractures in a level-1 trauma centre: Retrospective study of 414 patients over 10 years. Orthop Traumatol Surg Res.

[13] Peter RE (2015). Open reduction and internal fixation of osteoporotic acetabular fractures through the ilio-inguinal approach: use of buttress plates to control medial displacement of the quadrilateral surface. Injury.

[14] Sen RK, Saini G, Kadam S (2020). Anatomical quadrilateral plate for acetabulum fractures involving quadrilateral surface: a review. J Clin Orthop Trauma.

[15] Letournel E (1993). The treatment of acetabular fractures through the ilioinguinal approach. Clin Orthop Relat Res.

[16] Cole JD, Bolhofner BR (1994). Acetabular fracture fixation via a modified Stoppa limited intrapelvic approach. Description of operative technique and preliminary treatment results. Clin Orthop Relat Res.

[17] Bible JE, Choxi AA, Kadakia RJ (2014). Quantification of bony pelvic exposure through the modified Stoppa approach. J Orthop Trauma.

[18] Keel MJB, Ecker TM, Cullmann JL (2012). The pararectus approach for anterior intrapelvic management of acetabular fractures: an anatomical study and clinical evaluation. J Bone Jt Surg Br.

[19] Zha GC, Tulumuhan DM, Wang T (2020). A new internal fixation technique for acetabular fractures involving the quadrilateral plate. Orthop Traumatol Surg Res.

[20] Jeffcoat DM, Carroll EA, Huber FG (2012). Operative treatment of acetabular fractures in an older population through a limited ilioinguinal approach. J Orthop Trauma.

[21] Archdeacon MT, Kazemi N, Collinge C (2013). Treatment of protrusio fractures of the acetabulum in patients 70 years and older. J Orthop Trauma.

[22] Elnahal WA, Ward AJ, Acharya MR (2019). Does routine postoperative computerized tomography after acetabular fracture fixation affect management?. J Orthop Trauma.

